# Trace Mineral Source Influences Trace Mineral Solubility in Water and Mineral Binding Strength to Ruminal Digesta

**DOI:** 10.1007/s12011-024-04318-x

**Published:** 2024-07-30

**Authors:** Huey Yi Loh, Jerry W. Spears, Octavio Guimaraes, Alexandra C. Miller, Meghan P. Thorndyke, Tyler A. Thomas, Terry E. Engle

**Affiliations:** 1 Department of Animal Sciences, Colorado State University, Fort Collins, CO 80523 USA; 2 Department of Animal Science, North Carolina State University, Raleigh, NC 27695 USA

**Keywords:** Trace Mineral, Sulfate, Hydroxy, Solubility, Protozoa, Bacteria

## Abstract

Two experiments were conducted to examine the impact of trace mineral (TM) source on in vitro and in vivo solubility characteristics. Experiment 1: Hydroxy TM (HTM) and sulfate TM (STM) sources of Cu, Mn, and Zn were incubated separately in water for 24 h. Immediately after mixing, initial pH of each solution was greater (P < 0.03) for HTM compared to STM for all elements. Final pH tended to be greater for Cu (P = 0.09) and Zn (P = 0.07) from HTM compared to STM. Water solubility of Cu, Mn, and Zn from STM was greater (P < 0.01) than HTM sources. Experiment 2: Eight steers fitted with rumen cannula were blocked by body weight and randomly assigned to treatments. Treatments consisted of 10 mg Cu, 40 mg Mn, and 60 mg Zn/kg DM from either STM or HTM sources. Steers were individually fed a cracked corn-corn silage-based diet. Treatments were top-dressed daily. Rumen contents were collected at 0, 2, and 4 h post-feeding on d 1 and 14. On d 15, strained ruminal fluid and particle-associated microorganisms were obtained. Zinc was more tightly bound (P = 0.01) to the digesta in HTM-supplemented steers compared to STM on d 14. These data indicate that TM source influences pH and solubility of Cu, Mn, and Zn in water and may affect rumen soluble Cu concentrations and binding strength of Zn to solid digesta.

## Introduction

It has been well established that hydroxy trace minerals (HTM) sources of copper (Cu) and zinc (Zn) are relatively insoluble in water, whereas sulfate trace minerals (STM) sources of Cu and Zn are highly soluble in water [1–3]. When the pH of the aqueous solution is reduced below 4.0 with hydrochloric acid (HCl), Cu and Zn from both HTM and STM are highly soluble [1, 2]. In 2017, Faulkner and Weiss [4] were the first to report that Cu, manganese (Mn), and Zn from HTM improved fiber digestibility in lactating dairy cows compared to iso-amounts of supplemental Cu, Mn, and Zn from STM sources. High soluble concentrations of Cu and Zn in the ruminal environment can negatively impact rumen fermentation [5]. Therefore, the authors hypothesized that high rumen solubility of Cu, Mn, and Zn from STM sources may have impaired rumen microbial fermentation.

Recent research has confirmed the findings of Faulkner and Weiss [4]. Using a rumen pulse dose technique in canulated steers, Cu and Zn from HTM were less soluble in the rumen than Cu and Zn from STM sources when steers received a corn silage-steam-flaked based diet [6], a medium-quality hay diet [7], and a diet formulated for a high producing dairy cow [8]. Furthermore, Cu and Zn from HTM were less tightly bound to ruminal solid digesta than Cu and Zn from STM [6, 7]. Lactating dairy cows [9, 10] and steers [7, 8] supplemented with STM also had lower fiber digestion than those receiving HTM.

Solubility experiments comparing different trace mineral (TM) sources have used in vitro techniques or in vivo bolus dose models to investigate TM ruminal solubility. Limited research has investigated the impact of TM sources on these parameters through dietary inclusion. Therefore, the objectives of this study were to: 1) determine the effect of TM source (HTM vs STM) on pH and solubility of Cu, Mn, and Zn after incubation in water (pH 7.0); and 2) compare the effect of dietary TM source in vivo on: a) ruminal soluble TM concentrations, b) TM binding strength toward rumen digesta, and c) TM concentration in rumen bacteria and protozoa after feeding a diet supplemented with Cu, Mn, and Zn from STM or HTM sources. We hypothesized that STM forms of Cu, Mn, and Zn would have greater solubility in water, reduce water pH, have a greater solubility and binding strength to rumen digesta within the rumen, and bioaccumulate in protozoa and bacteria to a greater extent than HTM sources of Cu, Mn, and Zn.

## Materials and Methods

### Ethical Statement

Prior to the initiation of this experiment, all animal care, handling, and procedures described herein were approved by the Colorado State University Animal Care and Use Committee (IACUC approval #1555 and #3574).

### Mineral Sources and Composition

Two different sources of minerals were used throughout the entire experiment: 1) sulfate sources of trace minerals (STM): Cu sulfate pentahydrate (CuSO_4_·5H_2_O), Mn sulfate monohydrate (MnSO_4_·H_2_O), and Zn sulfate monohydrate (ZnSO_4_·H_2_O), and 2) hydroxy sources of trace minerals (HTM): IntelliBond C (Cu_2_(OH)_3_Cl_2_; Selko USA), IntelliBond M (Mn_2_(OH)_3_Cl; Selko USA), IntelliBond Z (Zn_5_(OH)_8_Cl_2_·H_2_O; Selko USA).

### Experiment 1 (In Vitro Solubility Study)

The solubility of Cu, Mn, and Zn from STM and HTM sources in this experiment was analyzed as described by Spears et al. [1] with slight modification. Briefly, approximately 0.025 g of Cu, Zn, and Mn from STM and HTM were brought to a final volume of 50 mL with deionized (DI) water in 50 mL conical tubes (n = 4 replicates/element/source, N = 24). Tubes were capped and swirled to mix the solution, and initial pH was measured. Samples were incubated for 24 h at 39 °C with agitation at 200 rpm. After 24 h, samples were filtered through ashless filter paper (Whatman plc, Maidstone, UK), and the final pH of the filtrates was measured. The filtrate was then analyzed for mineral concentration.

### Experiment 2

#### Animals and Diets

Eight crossbred Angus steers (509 kg ± 10 kg; approximately 1.5 years of age) fitted with ruminal cannula were utilized in this study. Before TM supplementation, all steers were transitioned to a diet formulated for a high-producing lactating dairy cow without Cu, Mn, and Zn supplementation for 28 d (Table 1). After the 28 d acclimation period, steers were stratified by body weight (BW) to treatments and individually housed where they received their respective trace mineral treatments: 10 mg Cu, 40 mg Mn, and 60 mg Zn/kg dry matter (DM) from either STM or HTM sources (n = 4/treatment). Each steer received approximately 11.6 kg of the basal diet (as-fed basis) per day for 14 d and individual intakes were recorded and measured daily. Dry distiller’s grain (DDG) was used as the carrier for the treatment. Treatment mixtures were top-dressed onto the feed and mixed thoroughly by hand after basal diet delivery. The basal diet contained 7, 57, and 35 mg/kg of DM of Cu, Mn, and Zn, respectively. Trace mineral supplements used in the experiment were analyzed and TM concentration ranged between 335–368 mg Cu, 1120–1580 mg Mn, and 2030–2340 mg Zn/kg DM of DDG, which was included as 2% of the diet. With the addition of TM supplementation, steers received approximately 16 mg Cu, 92 mg Mn, and 91 mg Zn/kg of DM.

**Table 1:** Ingredient and nutrient composition of the basal diet. (Experiment 2)

**Item**	
Ingredient, % dry matter basis	
Corn silage	27.64
Cracked corn	34.40
Distiller’s grains	12.78
Alfalfa hay	10.91
Wheat straw	8.44
Liquid supplement^1^	5.00
Limestone	0.67
Salt	0.17
Analyzed nutrient composition (DM basis)	
DM, % as fed	59.8
Crude protein, %	16.3
Acid detergent fiber, %	18.6
Neutral detergent fiber, %	31.1
Ether extract, %	6.4
NEg, Mcal/kg^2^	1.08
NEm, Mcal/kg^3^	1.73
Calcium, %	0.95
Magnesium, %	0.27
Phosphorus, %	0.31
Potassium, %	0.89
Sodium, %	0.21
Sulfur, %	0.20
Copper, mg/kg	6.97
Manganese, mg/kg	56.51
Zinc, mg/kg	34.52
Iron, mg/kg	111.26
Molybdenum, mg/kg	0.64

^1^Liquid supplement provided in a molasses suspension: 3.72% NPN (urea), 14.07% Calcium, 0.11% Phosphorus, 1.81% Potassium, 110,000 IU/kg vitamin A, 9.4 IU/kg vitamin E, and 440 g/metric ton of monensin (Rumensin 90, Elanco Animal Health, Greenfield, IN, USA)

^2^NEg = Net energy for gain

^3^NEm = Net energy for maintenance

Rumen contents were thoroughly mixed by hand and approximately 150 g rumen samples were collected in three 50 mL conical tubes from the geometric center of the rumen at 0, 2, and 4 h post-feeding on d 1 and d 14 of the experiment. The conical tubes were centrifuged at 1500 × *g* for 15 min immediately after collection and the supernatant was separated from the pellet. Both the supernatant and pellet were stored separately at -20 °C until analyzed for Cu, Mn, and Zn concentrations. Copper, Mn, and Zn concentrations within the supernatant were considered to be soluble whereas the Cu, Mn, and Zn concentrations in the digest were considered to be insoluble.

#### Dialysis

Dried digesta (pellet) samples from experiment 2 were exposed to dialysis for the investigation of the binding strength of minerals to the digesta. Dialysis tubing (regenerated cellulose, MWCO: 12-14kD, width: 45 mm, vol/length: 6.4 mL/cm; Spectrum™ Spectra/Por™, Fisher Scientific, Waltham, MA, USA) was prepared as described by Caldera et al. [6]. Briefly, dialysis tubing was cut into 15 cm segments and treated with 1mM ethylenediaminetetraacetate (EDTA) in 2% sodium bicarbonate solution to remove metal contamination. Treated dialysis tubing was rinsed with DI water, and stored in a solution containing 50% ethanol and 1 mM EDTA at 4 °C. Dialysis tubing was rinsed with DI water three times before use. Dialysis procedures were conducted as described by Jones et al. [11] and Guimaraes et al. [7] with slight modifications. Briefly, 0.25 g of samples were dialysis against 0.01 M EDTA in 0.05 M Tris (EDTA-Tris) buffer, adjusted to a pH of 6.8 using 6 M hydrochloric acid (HCl) and 10 M sodium hydroxide (NaOH). Samples were mixed with 10 mL of EDTA-Tris buffer and transferred into rinsed dialysis tubing. Samples were then dialyzed against 1.0 L of Tris–EDTA buffer for 16 h at 4 °C with continuous stirring. The buffer was replaced with another 1.0 L of EDTA-Tris buffer and the dialysis continued for another 6 h at 4 °C. After dialysis, samples were removed from the dialysis bags and placed into a pre-weighed acid-washed crucible. Samples were dried at 60 °C for 24 h, dry ashed at 600 °C or 12 h, reconstituted in 1.2 N HCl, and then analyzed for Cu, Mn, and Zn.

#### *Protozoa *and *Bacteria* Mineral Concentration

Approximately 250 g of rumen samples were collected from the geometric center of the rumen from each steer 2 h post-feeding 14 d after treatment initiation. Strained ruminal fluid (SRF) and particle-associated organisms (PAO) collection methods were adapted from methods described by Craig et al. [12] with slight modifications. Collected rumen samples were squeezed through 8 layers of cheesecloth into three 50 mL conical tubes to obtain SRF. Half of the remaining digesta (approximately 125 g) was placed in a bag and washed with phosphate-buffered saline (PBS) to obtain PAO. Protozoa and bacteria fractions were extracted through procedures described by Olubobokun et al. [13] and De Mulder et al. [14] with slight modifications. Briefly, SRF and PAO samples were centrifuged at 650 × g for 15 min to obtain protozoa pellet. The supernatant from the tubes was transferred into a new set of 50 mL conical tubes for bacteria extraction. The remaining pellet was washed again with PBS and centrifuged at 650 × g for 15 min. The pellet was the protozoa fraction of the samples and was analyzed for protozoal Cu, Mn, and Zn concentrations. The supernatant was then centrifuged at 25,000 × g for 30 min, the pellet obtained represented the bacteria fraction of the samples. The supernatant was discarded, and the bacteria pellet was washed with PBS and recentrifuged at 25,000 × g for 30 min. The supernatant was discarded, and the bacterial pellets were analyzed for bacterial Cu, Mn, and Zn concentration.

#### Mineral Analysis

Dry matter analysis was conducted on all solid samples prior to mineral analysis. All solid samples were analyzed using a dry ashing method according to the official methods of analysis [15]. In brief, approximately 1 g of sample was weighed into an acid-washed crucible and ashed in a Thermo-Fisher Thermolyne muffle furnace at 600 °C for 12 h. After ashing, samples were weighed and resuspended in a total of 5 mL of boiling 1.2 M HCl for mineral analysis. Samples were reconstituted with DI water to a total volume of 10 mL.

Liquid samples were digested using an open vessel digestion method adapted from Edgell [16] with slight modification. Briefly, 1 mL of samples were mixed with 70% nitric acid (Trace Element grade; ThermoFisher, Waltham, MA) at a 1:1 ratio. Sample-acid mixtures were left to react at room temperature under a fume hood for 10 min. After 10 min, samples-acid mixtures were placed into 70 °C water bath for 4 h with occasional swirling. Samples were removed from the water bath and allowed to cool to room temperature. After cooling, 1 mL of 30% hydrogen peroxide was added to the sample-acid mixture and allowed to react for 10 min. Samples were transferred back into the 70 °C water bath for 2 h. Samples were then diluted with DI water to fit within a linear range of a standard curve generated by linear regression of known TM concentrations and analyzed for Cu, Mn, and Zn via Atomic Absorption Spectroscopy (PinAAcle 500, PerkinElmer, Waltham, MA).

#### Statistical Analysis

All analyses in this experiment were performed for a randomized block design using R Statistical software version 4.3.2 and packages within R [17]. Initial pH, final pH, TM (Cu, Mn, Zn) concentration, and solubility percentage from experiment 1 and protozoal and bacterial TM concentrations from experiment 2 were analyzed using a student’s t-test. Trace mineral concentrations in the digesta and soluble fractions from experiment 2 and TM contractions of the digesta post-dialysis were analyzed using a linear mixed-effects model (lme4 ver. 1.1–32) [18, 19]. A three-way interaction between treatment x day x hour was analyzed using type III Analysis of Variance (car ver. 3.1–1) [20], with animal being the random effect in the statistical model. A two-way interaction (treatment x day) was investigated using a linear mixed-effects model with animal being the random variable. Least-squares means between treatment groups were estimated using emmeans package (ver. 1.8.5) [21]. For all response variables, a P-value of less than or equal to 0.05 was considered significant, and tendencies were determined with a P-value greater than 0.05 but less than or equal to 0.10.

## Results

### Experiment 1 (In Vitro Solubility Study)

The influence of trace mineral source on pH and TM solubility is shown in Table 2. Initial pH, at the time of mixing each TM source with water, was lesser for STM sources of Cu (P = 0.02), Mn (P = 0.006), and Zn (P = 0.002) compared to HTM sources of Cu, Mn, and Zn. Final pH following a 24-h incubation in DI water at 39 °C tended to be lesser for Cu (P = 0.09) and Zn (P = 0.07) from STM sources compared to Cu and Zn from HTM sources. Final pH was similar for both Mn sources (P = 0.16). At the end of the incubation, percent solubility of Cu, Mn, and Zn was greater (P < 0.01) for STM compared to HTM sources.

**Table 2:** Influence of trace mineral source on in vitro initial and final pH and trace mineral solubility after 24 h of incubation in deionized water at 39 °C. (Experiment 1)

Item	Treatment		SEM^3^	P <
	HTM^1^	STM^2^		
Initial pH				
Cu	8.16	5.54	0.5	0.03
Mn	7.60	6.10	0.3	0.01
Zn	7.32	6.17	0.2	0.01
Final pH				
Cu	5.82	4.18	0.3	0.10
Mn	6.75	4.98	0.4	0.16
Zn	6.16	4.55	0.33	0.07
Solubility (%)^4^				
Cu	1.34	70.35	13.1	0.01
Mn	13.88	93.75	15.1	0.01
Zn	3.13	98.23	17.4	0.01

^1^HTM = 0.025 g of Cu from CuOHCl, 0.025 g of Mn from MnOHCl, and 0.025 g of Zn from ZnOHCl were incubated in 50 mL of deionized water for 24 h at 39 °C

^2^STM = 0.016 g of Cu from CuSO_4_, 0.022 g of Mn from MnSO_4_, and 0.039 g of Zn from ZnSO_4_ were incubated in 50 mL of deionized water for 24 h at 39 °C

^3^SEM = standard error of the mean

^4^Solubility of each element following a 24-h incubation in deionized water at 39 °C

### Experiment 2

Table 3 describes the influence of TM source on soluble and insoluble Cu, Mn, and Zn concentrations in ruminal fluid and solid digesta fractions. There were no treatment x hour (P > 0.40) or treatment x day x hour (P > 0.30) interactions. Therefore, only main effects and the treatment x day interactions are shown. Data are compared by day within treatment and/or by treatment within day. There was a tendency (P = 0.087) for a treatment x day interaction for digesta Mn concentrations. Overall, Mn concentrations in digesta tended (P = 0.098) to be greater in STM supplemented steers on d 14 compared to HTM supplemented steers. Furthermore, Mn concentrations in digesta increased (P = 0.004) by 44.4% from day 1 to day 14 in STM supplemented steers. There was also a tendency (P = 0.055) for a treatment x day interaction for ruminal soluble Cu concentration. Ruminal soluble Cu concentrations increased (P = 0.002) by 27% from day 1 to day 14 in STM supplemented steers.

**Table 3:** Influence of trace mineral source on copper, manganese, and zinc concentrations of ruminal fluid and digesta fractions on day 1 and day 14 of the experiment. (Experiment 2)

Item^4^	Day				SEM^3^	P =			
	Day 1		Day 14						
	HTM^1^	STM^2^	HTM^1^	STM^2^		Trt	Day	Hour	Trt*Day
Digesta, mg/kg									
Cu	29.70	30.02	22.41	25.70	2.6	0.611	0.085	0.152	0.650
Mn	127.10	162.06^x^	141.21^a^	234.04^b,y^	21.7	0.217	0.013	0.924	0.087
Zn	153.91	153.98	187.62	210.20	20.1	0.724	0.058	0.757	0.625
Soluble, mg/L									
Cu	0.78	0.71^x^	0.83	0.98^y^	0.05	0.684	0.007	0.543	0.055
Mn	2.80	2.74	1.89	2.27	0.26	0.769	0.002	> 0.001	0.294
Zn	1.93	1.90	3.11	3.70	0.31	0.637	> 0.001	0.038	0.124

^1^HTM = 10 mg Cu/kg DM from CuOHCl; 40 mg Mn/ kg DM from MnOHCl; 60 mg Zn/kg DM from ZnOHCl

^2^STM = 10 mg Cu/kg DM from CuSO_4_; 40 mg Mn/ kg DM from MnSO_4_; 60 mg Zn/kg DM from ZnSO_4_

^3^ SEM = standard error of the mean

^4^The interaction of hour post-feeding (0, 2, and 4 h) or the interaction of hour post-feeding x treatment x day were not significant sources of variation in the model. Therefore, mineral concentrations were averaged for hour of sampling within a day

^a,^^b^Different superscripts indicate a significant difference between treatments within day; P-value < 0.10

^x^^,y^Different superscripts indicate a significant difference between days within the same treatment; P-value < 0.05

#### Dialysis

The influence of dialyzing rumen solid digesta against EDTA-Tris buffer on the remaining Cu, Mn, and Zn concentrations of rumen solid digesta on day 1 and day 14 of the experiment are shown in Table 4. There were no treatment x hour (P > 0.40) or treatment x day x hour (P > 0.10) interactions for any response variables. Therefore, data were compared by day within treatment and/or by treatment within day. A treatment x day interaction was detected (P = 0.024) for Zn remaining in the digesta post-dialysis. Zinc concentrations in rumen solid digesta were similar on d 1 for both treatments. On d 14, Zn concentration in the digesta of steers supplemented with HTM was greater (P = 0.01) than steers supplemented with STM. Post dialysis, the digesta collected from STM (P < 0.001) and HTM (P = 0.02) supplemented steers on day 1 had lesser Zn concentration compared to day 14.

**Table 4:** Influence of dialyzing rumen solid contents against EDTA-Tris buffer for 22 h on the remaining copper, manganese, and zinc content of rumen solid digesta collected, averaged across 0, 2, and 4 h post-feeding, on day 1 and day 14 of the experiment. (Experiment 2)

Item^4^	Day				SEM^3^	P =			
	Day 1		Day 14						
	HTM^1^	STM^2^	HTM^1^	STM^2^		Trt	Day	Hour	Trt*Day
Digesta, mg/kg									
Cu	15.92	14.51	22.08	10.94	3.5	0.181	0.755	0.447	0.245
Mn	17.80	18.63	21.96	25.15	1.6	0.579	0.016	0.026	0.560
Zn	9.61^x^	10.17^x^	20.06^a^^,^^y^	14.74^b,y^	1.5	0.102	> 0.001	0.402	0.024

^1^HTM = 10 mg Cu/kg DM from CuOHCl; 40 mg Mn/ kg DM from MnOHCl; 60 mg Zn/kg DM from ZnOHCl

^2^STM = 10 mg Cu/kg DM from CuSO_4_; 40 mg Mn/ kg DM from MnSO_4_; 60 mg Zn/kg DM from ZnSO_4_

^3^ SEM = standard error of the mean

^4^ The interaction of hour post-feeding (0, 2, and 4 h) or the interaction of hour post-feeding x treatment x day were not significant sources of variation in the model. Therefore, mineral concentrations were averaged for hour of sampling within a day

^a,^^b^Different superscripts indicate a significant difference between treatments within day; P-value < 0.05

^x^^,y^Different superscripts indicate a significant difference between days within the same treatment; P-value < 0.05

#### *Protozoa *and *Bacteria* mineral concentration

The concentration of Cu, Mn, and Zn in protozoal and bacterial fractions in SRF and PAO are shown in Table 5. Trace mineral source had no impact (P > 0.10) on Cu, Mn, and Zn concentrations of particle-associated or strained ruminal fluid bacteria or protozoa.

**Table 5:** Influence of trace mineral source on copper, manganese, and zinc concentrations (mg/kg DM) in protozoa and bacteria associated with the liquid and solid phases of ruminal contents. (Experiment 2)

Item	Treatment		SEM^3^	P <
	HTM^1^	STM^2^		
Protozoa				
SRF^4^				
Cu	82.91	95.42	4.1	0.149
Mn	401.71	358.97	42.8	0.654
Zn	484.17	458.72	18.2	0.666
PAO^5^				
Cu	100.08	101.01	3.5	0.906
Mn	330.76	337.35	27.1	0.915
Zn	602.86	580.25	26.1	0.589
Bacteria				
SRF^4^				
Cu	80.51	105.77	17.8	0.526
Mn	312.35	313.34	25.4	0.986
Zn	456.95	570.88	85.6	0.555
PAO^5^				
Cu	75.68	73.55	6.0	0.879
Mn	321.35	288.84	19.4	0.445
Zn	547.08	480.49	30.7	0.318

^1^HTM = Rumen contents were obtained from steers supplemented with 10 mg Cu/kg DM from CuOHCl; 40 mg Mn/ kg DM from MnOHCl; 60 mg Zn/kg DM from ZnOHCl for at least 14 days

^2^STM = Rumen contents were obtained from steers supplemented with 10 mg Cu/kg DM from CuSO_4_; 40 mg Mn/ kg DM from MnSO_4_; 60 mg Zn/kg DM from ZnSO_4_ for at least 14 days

^3^SEM = standard error of the mean

^4^Strained ruminal fluid

^5^Particle-associated microorganisms

## Discussion

The present study indicates that Cu, Mn, and Zn from HTM have a lower solubility in water when compared to STM sources. The solubility of Cu from the current study is similar to the findings from Spears et al. [1], where Cu from HTM was approximately 70% less soluble in water compared to Cu from STM. The solubility of Mn and Zn from the current study also follows the same trends as Cu, where Mn and Zn from HTM had a lower solubility compared to Mn and Zn from STM sources [2]. Water is a dipole molecule, with a strong electronegative oxygen that attracts electrons from hydrogens, causing the hydrogens to have a partial positive charge [22]. Due to the electrostatic forces of the dipole property of water, the sulfate molecule and the metal ion disassociate in water. In contrast, the covalent bond strength between hydroxychloride molecule and a particular element (e.g., Cu) within the crystalline matrix is greater than the electrostatic forces of water making the hydroxychloride-element molecule less soluble in water [3]. Manganese from HTM had a greater solubility when compared to Cu and Zn from HTM. The reason for the greater solubility is not well understood. Perhaps the reason for greater solubility in Mn from HTM in water compared to Cu and Zn from HTM sources is due to the difference in the number of protons between Mn (~ 25), Cu (~ 29), and Zn (~ 30). As the strength of a covalent bond depends on the extent of overlap of orbitals involved, the lower proton number in Mn may lead to a weaker positive force attraction towards the orbiting electrons compared to Cu and Zn, which may result in greater disassociation and solubility of HTM Mn in an aqueous environment.

When dissolved in water, STM sources of Cu, Mn, and Zn reduced pH to a greater extent than Cu, Mn, and Zn from HTM. This observed reduction in initial and final pH is due to the disassociation of metals (M; Cu, Mn, and Zn) and ${\text{SO}}_{4}^{2-}$ by the surrounding water molecules, which are dipolar molecules that can hydrolyze weak ionic bond within MSO_4_. The reaction between metals and water, ${\text{M}}^{2+}+2{\text{H}}_{2}\text{O }\leftrightarrow {\text{MOH}}^{+}+{\text{H}}_{3}{\text{O}}^{+}$, increases the concentration of H_3_O^+^, which leads to the reduction of pH. Initial pH values were greater for HTM vs STM of Cu, Mn, and Zn by 2.62, 1.51, and 1.15 pH units, respectively. After 24 h incubation at 39 °C, the solution containing Cu and Zn from HTM tended to have a greater final pH compared to STM. In general, the addition of Cu, Mn, and Zn from STM to water created a lower pH environment.

The rumen environment is very complex compared to controlled in vitro environments due to the presence of feed, microorganism populations, enzymes, ligands, etc. which can lead to mineral reduced mineral solubility and bioavailability. For example, high concentrations of soluble sulfur and molybdenum in the rumen can interact with soluble Cu and form insoluble Cu thiomolybdate complexes, which reduces Cu bioavailability [23, 24]. The diet used in the current study was low in TM antagonists (S, Mo, Fe, etc.). Solubility characteristics of Cu, Mn, and Zn may have differed if known TM antagonists were included in the diet. The in vitro rumen fermentation model can serve as a good indicator of what might be happening in the rumen, however, due to the buffering agents used, the lower liquid to TM ratio compared to estimated in vivo liquid to TM ratios, and the lack of removal of VFA from the system, interpretation of results is limited [25].

In the current study, Mn concentration in the digesta of steers fed HTM tended (P = 0.098) to be lower compared to STM on day 14, which may indicate that more Mn was released from HTM, but soluble Mn concentration was not different between treatments. A study conducted by Caldera et al. [6] observed a lesser soluble Mn concentration at 4 and 8 h post-dosing, and higher soluble Mn concentration in ruminal fluid 24 h post-dosing in HTM-supplemented steers compared to STM-supplemented steers. Guimaraes et al. [7] observed similar results when steers were fed medium-quality grass hay, where the soluble Mn concentration in the rumen was greater at 4 and 6 h post-dosing, but lesser at 18 h post-dosing in steers receiving STM than HTM. In addition, higher soluble Zn and Cu concentrations were observed in steer dosed with STM compared to HTM [6, 7]. In the present study, soluble concentrations of Zn and Cu were not affected by TM source. The potential reason that contributes to the difference between the current study and previous studies may be how the TM was delivered to the animals. In this study, TM supplementation was added to the total mixed ration, while studies conducted by Caldera et al. [6] and Guimaraes et al. [7] utilized direct bolus dosing of TM treatments into the rumen. Genther and Hansen [26] utilized dietary TM supplementation and observed lower rumen soluble Zn and Cu when expressed as a percent of whole rumen fluid concentration in HTM compared to STM supplemented cattle.

The binding strength of the mineral towards the digesta can also influence the bioavailability of TM. Although no difference was observed in Zn concentration in the digesta, Zn concentration in the digesta post-dialysis was greater in steers supplemented with HTM compared to STM on d 14, which indicates that Zn from HTM had a stronger binding strength toward the digesta compared to Zn from STM. The binding strength of Zn toward digesta changed over time. Post-dialysis, Zn concentration in the digesta was greater on day 14 compared to day 1 for both treatments, which may indicate that the binding strength increased over time. The amount of Cu and Mn not released following dialysis was not affected by TM source. In contrast, steers pulse dosed with HTM had a greater release of Cu and Zn from solid digesta than those dosed with STM following dialysis [6].

Mineral concentration in protozoal and bacterial fractions of rumen contents was not influenced by trace mineral source. The protozoal and bacterial fractionation procedures used in this study were adapted from Olubobokun et al. [13] and De Mulder et al. [14]. Olubobokun et al. [13] and De Mulder et al. [14] utilized diaminopimelic acid analysis, differential interference microscopy, and 16S sequencing to determine the purity of their protozoa and bacteria fractions. The authors reported that after fractionation of ruminal contents via filtration and centrifugation, the protozoal fraction contained bacteria associated with small feed particles. Although not measured in the current study, it is likely that the protozoa fraction contained bacteria, which most likely contributed to the Cu, Mn, and Zn concentrations of the protozoal fraction. A study conducted by Gresakova et al. [27] observed a higher Mn bacterial concentration in sheep fed Mn glycine hydrate compared to Mn sulfate. They also observed variations in Cu, Mn, and Zn concentrations in rumen protozoal and bacterial fractions when different Mn concentrations were supplemented. A potential reason for the discrepancies between studies may be due to the length of the feeding period. In the current experiment, the feeding period was 14 days compared to the study conducted by Gresakova et al. [27], which was 16 weeks. A longer feeding period may be required to change the mineral concentration in the protozoa and bacteria population residing in the rumen.

## Conclusion

Results from the in-vitro study of this experiment indicate that HTM is less soluble than STM in water and that STM Cu, Mn, and Zn decrease the solute pH at neutral pH environments. Hydroxy TM also contributed to a higher initial pH for all elements and tended to have a higher final 24 h pH for Zn and Cu compared to STM. Dietary intake of different mineral sources can potentially influence Mn solubility and Zn binding strength toward the digesta. This study did not observe changes in TM concentration in the protozoal and bacterial fraction of rumen fluid. Longer term studies to investigate the interaction of TM source with rumen microorganisms are warranted.

## Data Availability

The datasets generated during and/or analyzed during the current study are available upon request from the corresponding authors.
